# Mini Cleanroom for the Manufacture of Advanced Therapy Medicinal Products (ATMP): Bioengineered Corneal Epithelium

**DOI:** 10.3390/pharmaceutics13081282

**Published:** 2021-08-17

**Authors:** Silvia Berisa-Prado, Natalia Vázquez, Manuel Chacón, Mairobi Persinal-Medina, Sergio Alonso-Alonso, Begoña Baamonde, José F. Alfonso, Luis Fernández-Vega-Cueto, Jesús Merayo-Lloves, Álvaro Meana

**Affiliations:** 1 Instituto Universitario Fernández-Vega, Fundación de Investigación Oftalmológica, Universidad de Oviedo, 33012 Oviedo, Spain; sberisa@unav.es (S.B.-P.); natalia.vazquez@fio.as (N.V.); m.chacon@fio.as (M.C.); mairobimedina@gmail.com (M.P.-M.); sergio.alonso@fio.as (S.A.-A.); bbaamonde@yahoo.es (B.B.); j.alfonso@fernandez-vega.com (J.F.A.); lfvc@fernandez-vega.com (L.F.-V.-C.); merayo@fio.as (J.M.-L.); 2 Instituto de Investigación Sanitaria del Principado de Asturias (ISPA), 33011 Oviedo, Spain; 3 Centro de Investigación Biomédica en Red en Enfermedades Raras (CIBERER) (U714), ISCII, 28029 Madrid, Spain

**Keywords:** advanced therapy medicinal products, ophthalmology, hospital pharmacy

## Abstract

Among several requirements for the manufacture of Advanced Therapy Medicinal Products (ATMP) are: following the guidelines of a pharmaceutical quality system, complying with Good Manufacturing Practice (GMP) and access to a cleanroom fulfilling strict environmental conditions (Class A work area and Class B environment). This makes ATMP expensive. Moreover, the production of many of these therapeutic products may also be unprofitable, as in most cases their use is limited to a few patients and to a single batch per manufacturing unit. To reduce costs, ATMP may be produced in a scaled-down system isolated from the external environment (isolator), allowing for placement of this facility in a Class D environment, which is much more permissive and less costly. In this work, we confirm that it is possible to manufacture bioengineered corneal epithelium inside an isolator while fulfilling all the safety assurance standards at an affordable cost for patients. This small-scale ultra-clean working environment complies with GMP guidelines and could be a solution for the high costs associated with conventional cleanroom ATMP production.

## 1. Introduction

Advanced Therapy Medicinal Products (ATMP) for human use include gene therapy, cell therapy and tissue engineering products. These ATMP offer treatment options for diseases that today lack effective medication. An example is Limbal Stem Cell Deficiency (LSCD), an orphan disease (171,673), for which traditional keratoplasty leads to a poor prognosis as there are few Limbal Stem Cells (LSC) to renew the corneal epithelial surface. The in vitro expansion of LSC has shown satisfactory results, and Holoclar^®^ (Chiesi, Parma, Italy) is the first ATMP used in Europe to treat LSCD after the European Medicine Agency (EMA) granted it marketing authorization [1]. As mouse cells and bovine serum are employed in the production of this drug, it is contraindicated for use in patients who are hypersensitive to xenogeneic proteins [2].

Recently, the Spanish Fundación de Investigación Oftalmológica developed a simple culture method for the in vitro expansion of LSC avoiding xenogeneic components, in which Plasma Rich in Growth Factors (PRGF) is used as the only culture medium supplement and to construct a PRGF membrane to culture and transplant the LSC [3]. Under current European legislation, this form of therapy is defined as an ATMP.

In Europe, ATMP are legislated by Regulation (EC) N° 1394/2007 of the European Parliament and of the Council [4]. For ATMP produced for use in a single hospitalized patient, this regulation contemplates “hospital exemption”. In Spain, the Real Decreto 477/2014 regulates authorization of the non-industrial manufacture of ATMP, requiring compliance with Good Manufacturing Practice (GMP) and with the medicinal products’ law whereby a cleanroom facility is a production requirement [5,6,7].

Traditional cleanrooms are typically containment rooms where the air is filtered and recirculated through a High Efficiency Particle Arresting (HEPA) filtration system. These installations, containing all the necessary equipment for the production of an ATMP, are expensive to build and maintain. To reduce the costs of traditional cleanrooms and also allowing for their customization, new modular cleanrooms have recently emerged. However, although maintenance costs are lower, the price of their design is even higher than for traditional cleanrooms. These requirements make the manufacture of ATMP expensive, especially as they themselves are unprofitable to produce because their use is limited to few patients and to a single batch per manufacturing unit. An alternative that would reduce costs is to produce the ATMP in a system isolated from the external environment, like an isolator, and to place this system in a Class D environment, which is much more permissive and less costly.

The aim of this work was to design a reduced size, low-cost cleanroom installation that complies with GMP standards for the production of an ophthalmologic ATMP, bioengineered corneal epithelium. Together with our Pharmacy Service, in the present study we developed a mini cleanroom with all the equipment necessary to manufacture a bioengineered corneal epithelium (PRGF scaffold with cultured LSC on top). This ultra-clean workstation was then used to prepare this ATMP which complies with all safety assurance regulations and is affordable for patients.

## 2. Materials and Methods

### 2.1. Mini Cleanroom Design

In accordance with GMP, the following user requirement specifications were established:

- Positive pressure Grade A isolator (laminar flow between 0.25 m/s and 0.50 m/s with 90% recirculation of air, International Organization for Standardization (ISO) 4 [6,8]
- Dimensions that allow the installation of all the equipment necessary for production (cell incubator, centrifuge and phase-contrast microscope with monitor)
- Lateral Special Airlock System (SAS) with double door lock
- Universal serial bus (USB), High-Definition Multimedia Interface (HDMI) and alternative current/discontinuous current (AC/DC) connectors

### 2.2. Qualification of the Mini Cleanroom

The mini cleanroom was required to pass through all stages of qualification planning: Installation Qualification (IQ), Operational Qualification (OQ) and Performance Qualification (PQ) included in the validation master plan. Qualifications were conducted in accordance with predetermined and approved qualification protocols. Previously, the area where the mini cleanroom was located was also qualified.

#### 2.2.1. Installation Qualification (IQ)

IQ was carried out according to the IQ protocol which contains all the required details:

- Isolator model and serial number
- Details of the supplier and manufacturer
- Installation date
- Mini cleanroom designed in accordance with design specification and requirements
- Verification of isolator elements
- Operating and working instructions, maintenance requirements and certificate of OQ at source

During installation, standard operational procedures (SOPs) with the process for operations, maintenance and cleaning of the mini cleanroom were drawn up.

#### 2.2.2. Operational Qualification (OQ)

Complex operational parameters were qualified by the supplier (TDI, Madrid, Spain). OQ was performed “at rest” according to the OQ protocol which contains all the required details and the limits established (Table 1, Table 2 and Table 3):

- Particle counts for cleanroom air quality classification according to ISO 14644-1 [8]
- Airflow velocity measurement
- Testing installed HEPA filter
- Air flow distribution test
- Weekly environmental monitoring (3 weeks) including air sample on individual tryptic soy and Sabouraud’s dextrose agar settling plates (Biomérieux, Marcy-l’Étoile, France) exposed for less than 4 h. Surface samples including isolator walls and equipment
- Weekly counting particle counts (3 weeks) using an HHPC 3+ counter (0.3 µm) (HACH^®^, London, OR, Canada)
- Weekly monitoring of control panel parameters ensuring that alert and action limits are not exceeded (3 weeks)
- Weekly cleaning of the mini cleanroom as per SOP (3 weeks)

#### 2.2.3. Performance Qualification (PQ)

PQ was carried out at rest or in operation according to a PQ protocol which contains all the required details:

- Cleaning of mini cleanroom for four months
- Environmental monitoring of mini cleanroom for four months
- Particle count monitoring of mini cleanroom for four months
- Control panel monitoring for four months

### 2.3. Quality Program According to GMP Procedures

A quality program was developed in accordance with the Real Decreto 477/2014 and GMP Part IV [5,6] to ensure:

- Adequate staff training
- Appropriate facilities and equipment for intended use with adequate cleaning and maintenance
- A document system with appropriate specifications of the materials used in the production process as well as of the finished product which comply with the defined specifications, keeping the appropriate records.
- A clearly defined production process
- A quality control system independent of the production system
- A self-assessment system
- Quality defects and process deviations are identified, causes are investigated and corrective and/or preventive measures are taken
- Traceability of the ATMP manufactured and the materials used in the production process

### 2.4. ATMP Production in the Mini Cleanroom

Production was transferred from the basic research laboratory to the mini cleanroom. The first step was to validate the aseptic technique by all the staff involved in ATMP production. Briefly, the steps described in the production guide were simulated using trypticase soy growth (TSG) media. Next, TSG media were incubated for 14 days at 30–37 °C. The aseptic technique was validated if no microbiological contamination was observed. Once validated, three production batches were prepared in the mini cleanroom according to written SOPs:

#### 2.4.1. Preparing the Culture Medium and PRGF Membranes for the Production of Bioengineered Corneal Epithelium

Blood was collected from healthy volunteers after explaining to them the nature and possible consequences of the study and obtaining their written consent. The study protocol adhered to the principles of the Declaration of Helsinki (Protocol Code: 2020.050; Comité de Ética de la Investigación del Principado de Asturias; 23 March 2020). Blood samples from each volunteer were processed according to the method described by Anitua et al. [9,10]. Briefly, 81 mL of blood were collected into 9 mL tubes containing 3.8% (*wt*/*v*) sodium citrate, and the tubes were centrifuged at 580× *g* for 8 min at room temperature.

**Figure 1:**
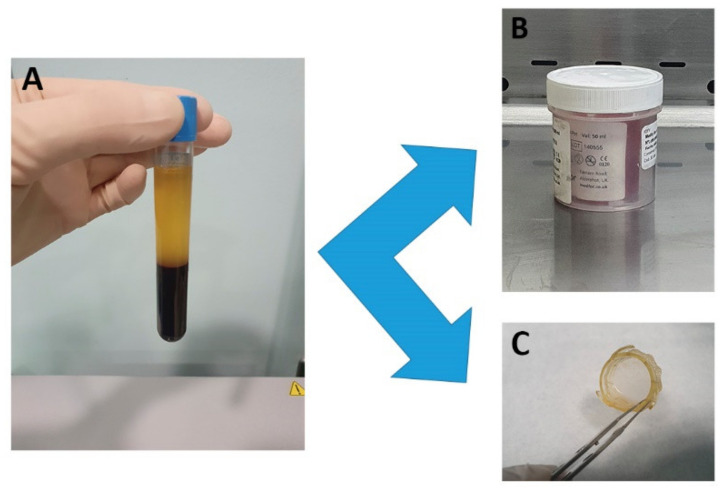
Human plasma (**A**) is used both as supplement (PRGF) for the culture medium (**B**) and as a scaffold (PRGF membrane) (**C**) for culturing and delivering LSC. PRGF: Plasma Rich in Growth Factors; LSC: Limbal Stem Cell.

Plasma was used as the only supplement in the culture medium and as a fibrin scaffold for culturing and delivering LSC [3] (Figure 1). Briefly, the plasma was drawn off, avoiding the buffy coat, incubated with 50 µL/mL of 10% CaCl_2_ (B. Braun Medical, Barcelona, Spain) at 37 °C for 30 min and the released supernatant (PRGF) was collected:

Culture medium: the culture medium was a 2:1 mixture of Dulbecco’s Modified Eagle’s Medium (DMEM; Thermo Fisher Scientific, Waltham, MA, USA) and Ham F12 (Thermo Fisher Scientific) supplemented with 10% PRGF. Once prepared, it was filtered through a 0.20 µm polyethersulfone membrane (Thermo Fisher Scientific), aliquoted and stored at 4 °C in a double container until use.

PRGF membranes: 10 mL aliquots of recovered plasma were incubated with 500 µL of 10% CaCl_2_ in a 60 × 15 mm cell culture Petri dish at 37 °C for at least 30 min. Once a gel had formed, PRGF membranes were obtained by flattening for 30 s using a 100 µm fibrin membrane shaper (BTI, Vitoria, Spain). The PRGF membranes obtained were placed onto a CellCrown™ insert (Scaffdex, Tampere, Finland) to improve handling and stored with 4 mL of PRGF at 4 °C in a double container until use.

#### 2.4.2. Establishing the Primary Culture

Limbus explants from cadaveric donors were obtained from our local Tissue Bank, Centro Comunitario de Sangre y Tejidos de Asturias (CCST) according to Regulation (EC) N° 23/2004 [11]. Briefly, the limbal region was carefully dissected according to its anatomical position and then cut into thirteen pieces of approximately 1–2 mm^2^ that were stored at 4 °C in an Eusol bottle (Alchimia, Ponte San Nicolò, Italy) until use. In the mini cleanroom, 12 tissue pieces were placed in the wells of a 12-well culture plate with culture medium and cultured at 37 °C, in a 5% CO_2_ atmosphere in an incubator.

#### 2.4.3. Medium Changes

A few drops of culture medium were added every day until the LSC began to proliferate from the explant. Hereafter, the culture medium was changed twice a week.

#### 2.4.4. Culturing LSC on the PRGF Membranes to Prepare the Bioengineered Corneal Epithelium

Cultures of LSC were examined every day using the phase-contrast microscope installed in the mini cleanroom. Once confluent, photographs were taken and one of the well cultures was fixed with methanol for 10 min for cytokeratin (CK) with high molecular weight (Ref: M0630, Agilent Dako, Santa Clara, CA, USA) and the transcription factor p63-alpha (Ref: 4892S, Cell Signaling Technology, Danvers, MA, USA) immunofluorescence in order to identify corneal epithelial cells and LSC respectively. The rest of the well cultures were trypsinized, followed by counting cells with a hemocytometer, seeding them (>300,000 cells per membrane) onto a PRGF membrane placed in the well of 6-well culture plate with culture medium and culturing them for 72 h in the same culture medium supplemented with 1.5 mg/mL tranexamic acid (Amchafibrin^®^, Meda Pharma, Madrid, Spain).

Immunofluorescence analysis was performed in the microscopy facility of the Fundación de Investigación Oftalmológica. Briefly, fixed cells were rinsed with Phosphate Buffered Saline (PBS) solution twice for 10 min and permeabilized in a PBS solution containing 0.3% Triton X-100 for another 5 min. Next, the samples were incubated at 4 °C overnight with primary antibodies (p63-alpha, 1:100; CK, 1:40) containing 10% normal goat serum (Life Technologies, Carlsbad, CA, USA) as a blocking agent. Immunolabeled cells were visualized by indirect immunocytochemistry and stained with 4′,6-DiAmidino-2-PhenylIndole (DAPI) to visualize nuclei. Samples were examined in a Leica DM6000B fluorescence microscope (Leica, Wetzlar, Germany) and five photos of random fields were captured using a Leica DFC310FX camera at 20× magnification. In the photographs, cells staining positive for p63-alpha out of the total of DAPI positive cells (%) were counted using ImageJ software (NIH, Bethesda, MA, USA).

#### 2.4.5. Final Release of ATMP

After 72 h of culture, the ATMP was packaged in its final primary and secondary containers, labeled and released once subjected to all the quality control checks.

#### 2.4.6. Quality Control

##### Process Quality Control

To identify the critical manufacturing steps, a risk manual was prepared including a Hazard Analysis and Critical Control Points (HACCP). In the critical manufacturing steps, viable and non-viable particle counts were monitored.

##### Product Quality Control

For the ATMP, the quality controls steps were:

- Examine the morphology by phase-contrast microscopy and CK immunofluorescence and determine the percentage of p63-alpha positive cells (LSC) in the primary culture using the immunofluorescence technique described above
- Count the number of cells of five photos of random fields at 20x magnification in the ATMP by DAPI staining
- Through p63-alpha immunofluorescence, confirm the presence of the LSC in the final ATMP
- Microbiological control of the manufactured ATMP consisting of an initial (plasma and limbal tissue), intermediate (supernatant) and final microbiological analysis by an external laboratory according to Chapter 2.6.1 of the European Pharmacopoeia [12], which previously had validated the growth promotion of the culture medium (aerobes, anaerobes, mycoplasma and endotoxins).

## 3. Results

### 3.1. Mini Cleanroom Design

The mini cleanroom consisted of an isolator of positive pressure ISO 4 (AISLAISO4-PP-1800^®^, TDI, Madrid, Spain) of exterior dimensions 1880 × 780 × 1620 cm, equipped with a lateral SAS with an independent ultraviolet (UV) sterilization system and a double-door lock along with a gas outlet with an electronically controlled valve and USB, HDMI and AC/DC connectors. To ensure air purity, the isolator was fitted with HEPA H14 filters (TDI) with an efficiency of 99.995% according to EN1822 regulation [13], which provides laminar flow of 0.25 m/s to 0.50 m/s with 90% air recirculation.

**Figure 2:**
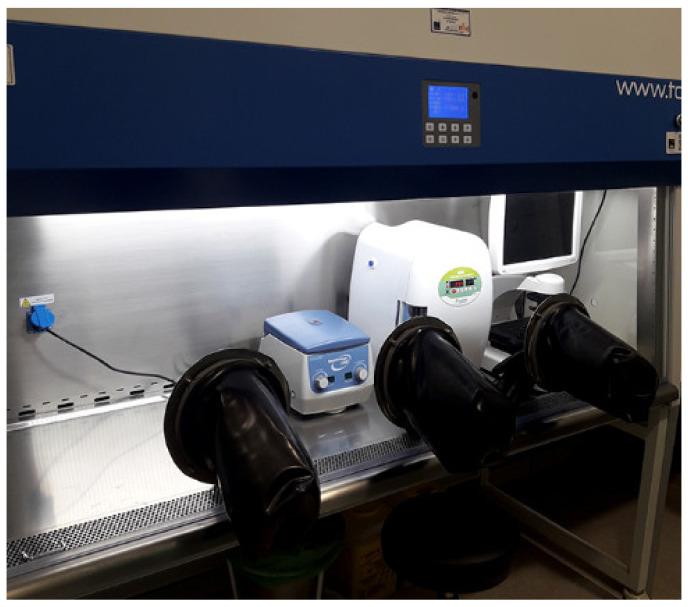
Mini cleanroom.

The equipment needed to produce the ATMP installed inside the isolator facility consisted of a small portable CO_2_ incubator (Minicell NB203M, N-Biotek, Gyeonggi-do, Korea), a phase-contrast microscope (EVOX^®^ XL Cell Imaging System, Thermo Fisher Scientific) and a compact centrifuge (Spectrafuge 6C, Labnet, Madrid, Spain) (Figure 2).

The mini cleanroom workstation was set up in a qualified Grade C area authorized for the elaboration of sterile products within the Pharmacy Service of the Instituto Oftalmológico Fernández-Vega.

### 3.2. Qualification of Mini Cleanroom

#### 3.2.1. Installation Qualification (IQ)

**Figure 3:**
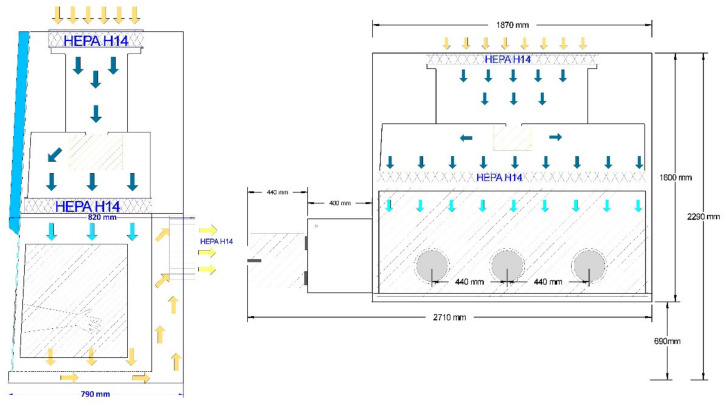
Front and side view of the isolator filtration system. HEPA: High-Efficiency Particle Arresting.

The mini cleanroom complied with user requirement specifications. Below we provide drawings of the mini cleanroom layout and air handling units (Figure 3).

In addition, it was checked that finishes were smooth, defect-free, and without uncleanable recesses, that SAS doors were interlocked to prevent both doors being opened simultaneously, and that panel controls were accessible. Finally, we made sure that instructions and certificates of OQ were available at the source.

#### 3.2.2. Operational Qualification (OQ)

Testing by the external supplier indicated that the mini cleanroom complied with the required ISO 4 classification in terms of non-viable particle counts at rest, having positive pressure and being airtight. In addition, the results of the laminar flow test performed at eight points in the isolator indicated no areas of turbulence in compliance with the standards presented in ISO 14644-1 [8]. Environmental conditions, non-viable particle counts, and control panel parameters were within the allowed limits over the 3 weeks of weekly monitoring. Finally, cleaning was easy to carry out (Table 4).

#### 3.2.3. Performance Qualification (PQ)

Control panel parameters and viable and non-viable particle counts at rest and in operation were within permissible limits over the four months of continuous monitoring (Table 5).

Moreover, all the equipment and instruments used in the manufacture of the ATMP were also qualified and calibrated.

### 3.3. Quality Program according to GMP Procedures

The quality program implemented covers all aspects of production, from starting materials, premises and equipment, through to staff training and personal hygiene. For this purpose, SOPs were developed providing documented proof that correct procedures were consistently followed at each step of the manufacturing process.

SOPs were classified into:

- General SOPs: for general operations and activities related to the procedure to be carried out
- Control SOPs: describing the operations to carry out for environmental controls of the isolator and quality control of the ATMP
- Production SOPs: describing operations directly related to ATMP production
- Equipment operating and maintenance SOPs: describing the operations to be carried out for proper operation and maintenance of the different devices
- Qualification SOPs: describing the operations that must be carried out to confirm that the qualified devices comply with the manufacturer instructions and with GMP
- Validation SOPs: describing the operations that must be carried out for documented evidence that a process meets the specifications and/or quality characteristics defined

Each SOP consists of the following sections: the purpose of the SOP, the staff responsible for complying with the procedure, the description of the procedure, the references and records generated by the SOP or other information that it is considered of interest.

Moreover, the validation master plan, the risk manual with a HACCP, the stability study and the technical sheet were also written.

### 3.4. ATMP Production in the Mini Cleanroom

Three batches of our ATMP were produced in the mini cleanroom following the protocol described above.

#### 3.4.1. Process Quality Controls

The HACCP established three critical manufacturing steps: preparation of the culture medium and PRGF membranes and establishing the primary LSC culture; culture of LSC on PRGF membrane; and final release of ATMP. In these steps, we confirmed that viable and non-viable particle counts in the three batches were within permitted limits (Table 6).

#### 3.4.2. Product Quality Control

**Figure 4:**
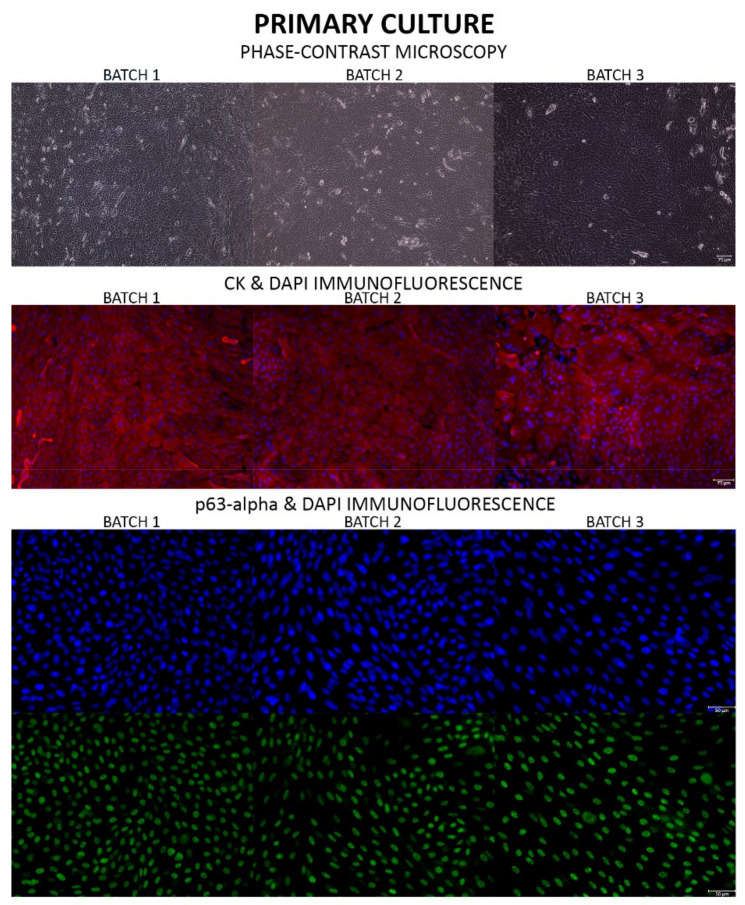
Phase-contrast microscopy (Scale: 75 µm) and immunofluorescence analysis of the LSC primary cultures in which CK positive LSC appear red (Scale: 75 µm), p63-alpha positive LSC appear green (Scale: 50 µm) and DAPI appears blue. LSC: Limbal Stem Cell; CK: cytokeratin.

In the three batches prepared, LSC began to migrate from the limbal explant onto the culture plate after 4–8 days of culture and a monolayer of LSC, flat and round in shape, was observed on confluent culture plates (day 15) by phase-contrast microscopy (Figure 4). Immunofluorescence analysis (Figure 4) indicated that primary LSC cultures were CK and p63-alpha positive: the percentage of p63-alpha positive cells in the primary LSC culture (Table 7) was 76.38 ± 1.49% (72.03–80.31%), which is slightly higher than the limits established (63.00 ± 1.93%) in prior work [3].

**Figure 5:**
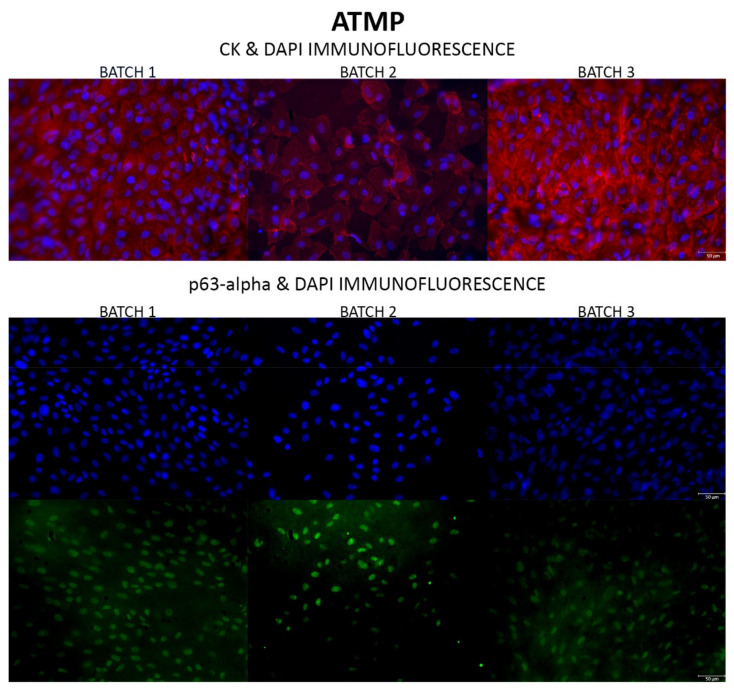
Immunofluorescence analysis of ATMP in which CK positive LSC appear red, p63-alpha positive LSC appear green and DAPI appears blue (Scale: 50 µm). ATMP: Advanced Therapy Medicinal Products; CK: cytokeratin; LSC: Limbal Stem Cell.

DAPI analysis of the manufactured ATMP revealed an LSC count (Table 7) of 139,306 ± 10,160 cells/cm^2^ (104.446–171.504 cells/cm^2^), well within the number of cells established for the ATMP in the technical sheet (79,000–316,000 cell/cm^2^). Moreover, immunofluorescence analysis of the manufactured ATMP (Figure 5) indicated that ATMP were CK and p63-alpha positive: the percentage of p63-alpha positive cells in the ATMP (Table 7) was 57.27 ± 5.91% (44.55–82.37%).

Finally, initial (plasma), intermediate (supernatant) and final microbiological checks of the manufactured ATMP (Table 7) indicated no microbiological contamination in any of the batches prepared.

## 4. Discussion

ATMP are drugs for human use designed to treat diseases that are refractory to currently marketed drugs. The regulation applicable to these drugs is European Regulation (EC) N° 1394/2007 of the European Parliament and of the Council of 13 November 2007 on ATMP [4], which applies to centralized authorization by the EMA.

ATMP must meet two main requirements to be commercialized and approved by regulatory agencies: confirmed safety assurance and efficacy of the therapy [14], and consistent and rigorous manufacturing with well-defined quality [15] while maintaining economic profitability [16]. Due to the enormous manufacturing challenges that have to be overcome for approval, only two ocular ATMP have been approved in Europe: Luxturna^®^ (Spark Therapeutics, Philadelphia, PA, USA), an ATMP for the treatment of inherited retinal dystrophy [17], and Holoclar^®^, an ATMP for the treatment of moderate to severe LSCD caused by burns [18,19].

Inherited retinal dystrophy and LSCD are considered rare diseases. In Europe, a rare disease is one that affects 1 person per 2000 and European regulation contemplates “hospital exemption” to manufacture one ATMP for the treatment of a single patient, within the framework of a hospital institution responsible for ATMP manufacture following the strict quality conditions required for conventional drugs (GMP) [6].

In Spain, the Real Decreto 477/2014 [5] is applicable to ATMP for human use which comply with “hospital exemption”. These products are occasionally prepared in accordance with specific quality standards, and used in a hospital institution under the exclusive professional responsibility of a registered physician. They are also required to correspond to an individual medical prescription for a custom-made product intended for a single patient, as defined in Regulation (EC) N° 1394/2007 [4]. The Spanish Agency for Medicines and Health Products (AEMPS) confirms that these ATMP satisfy criteria of quality, safety, efficacy, identification and information [4,5]. Currently, there are two approved drugs according to this Real Decreto [20]: ARI-0001 cellular dispersion for perfusion, a CAR-T cell therapy for the treatment of acute lymphoblastic leukemia (Hospital Clínic de Barcelona, Cataluña, Spain) and autologous bone marrow stem mesenchymal cells, an ATMP developed to treat spinal injury (Hospital Universitario Puerta del Hierro, Madrid, Spain).

The manufacture of these ATMP require authorization by the AEMPS, which includes being approved for complying with GMP. Conventional cleanrooms are ideal for this purpose, yet with the limitation of both installation and maintenance costs, making ATMP treatments inaccessible for most healthcare systems. In ophthalmology, where an ATMP may have a volume of less than 1 mL or in the case of an epithelial corneal lamella graft, a diameter of less than 3 cm, the use of a conventional cleanroom of several square meters is even more unprofitable. This is especially true when manufacturing a small number of batches as in the case of an ATMP manufactured for the treatment of a rare disease.

The use of low-cost infrastructures and simple maintenance, complying with all GMP requirements, is a feasible alternative to conventional cleanrooms. Sophisticated designs currently exist and have been adopted by the pharmaceutical industry to produce cells and ATMP. An example is the Integrated Cell Processing Workstation^®^, Isocell pro^®^ (Tecomak, Tonbrige, UK) and Cell Processing Isolator^®^ (ESCO, Barnsley, UK). This is an integrated system for GMP-compliant processing and preparation of regenerative stem cell and cell therapies. However, these new technologies have been developed for several batches and not for a single batch for one patient. This means that even these infrastructures end up being costly as production is limited.

In previous works, we developed a culture method for the in vitro expansion of LSC, in which PRGF is used as the only culture medium supplement and to construct a PRGF membrane to culture and transplant the LSC [3]. Moreover, we demonstrated that PRGF membranes with autologous or heterologous rabbit LSC were able to restore the corneal surface in a rabbit alkali-burn model [21].

In the present work, we designed a mini cleanroom facility to transfer ATMP production in the research laboratory to a mini cleanroom, of lower installation and maintenance costs and complying with GMP. In this facility, production is carried out in a space isolated from the outside environment, such that LSC can be expanded and monitored throughout the production process. Protocol qualification revealed that the mini cleanroom proposed here complies with the required ISO 4 classification according to ISO 14644-1, had positive pressure, was airtight, had laminar airflow without turbulence, and environmental and control panel parameters were within allowed limits. The benefits of isolators over conventional cleanrooms such as air pressure differential, small footprint and relative ease of decontamination make a good case for their superior sterility assurance potential.

For our proposal, we adapted a quality program to this special workstation, or mini cleanroom, that enables the production of an ATMP with the requirements of quality, safety assurance and efficacy. The work scheme complies with current regulations regarding ATMP requirements. While with this facility we were able to produce an ATMP saving on installation and maintenance costs, the quality program developed required the same large amount of documentation as for a conventional cleanroom.

The functionality of the mini cleanroom was confirmed through the production of three batches of our ATMP. Cumulatively, these batches revealed that the ATMP had several cells per cm^2^ within the limits allowed in the Holoclar^®^ technical sheet approved by the EMA [19]. Moreover, the percentage of p63-alpha positive cells in the primary LSC culture was slightly higher than the limit established in our prior work [3]. Since LSC cultured onto PRGF membranes are difficult to see by phase-contrast microscopy we demonstrated optimal LSC morphology by CK immunofluorescence and we confirmed the presence of a high level of p63-alpha positive cells in the ATMP which sustains the regenerative potential of LSC [22]. This guarantees the efficacy of our ATMP as these cells will proliferate after transplantation and regenerate the ocular surface. In addition, this criterion ensures the reproducibility of our production process.

Despite the described benefits of this affordable installation, because of the limited working space, staff training is required. This could be a drawback for transferring this installation to other specialties that require larger-scale production. However, consumables, cleaning and validation costs, along with operator time and working conditions, are improved in the mini cleanroom approach, thus contributing to the critical goal of procedure optimization and reduction of final product costs. Our design could help with the development of new ophthalmology products such as gene therapy agents for retinal diseases or engineered lamellar endothelial tissue. It should be noted that for many patients needing a cornea transplant, there is no donor cornea available, especially in countries where tissue banks are scarce or lacking. The possibility of new treatment options for corneal transplants manufactured in low-cost laboratories may be an important step forward to treating some causes of blindness and facilitating access to treatments to many patients. Facilities such as that proposed here could be a starting point for new therapies within reach of the few patients who suffer from a rare ophthalmologic disease. Furthermore, this mini cleanroom facility could also be applied to the manufacturing of other types of ATMP which require GMP-quality manufacturing.

## 5. Conclusions

We here propose an innovative installation, a mini cleanroom, or small-scale GMP-compliant ultra-clean workstation, as a solution for the high costs associated with cleanroom facilities generally used for ATMP production. This design could enable small laboratories to prepare relatively low-budget therapeutic products, thus reaching a greater number of patients.

## Figures and Tables

**Table 1:** Established alert and action limits for control panel parameters.

	Alert	Action
Exhaust filter	15–20 Pa	<15 Pa
Downflow filter	>120 Pa	>140 Pa
Inflow	<0.28 m/s or >0.47 m/s	<0.20 m/s or >0.55 m/s
Temperature	>25 °C	>30 °C
Humidity	>55%	>60%

**Table 2:** Non-viable particle counts. ISO: International Organization for Standardization.

ISO 14644-1 Classification						
Concentration of Particles per Cubic Meter of Air						
CLASS	≥0.1 µm	≥0.2 µm	≥0.3 µm	≥0.5 µm	≥1 µm	≥5 µm
ISO 1	10	2	-	-	-	-
ISO 2	100	24	10	4	-	-
ISO 3	1000	237	102	35	8	-
ISO 4	10,000	2370	1020	352	83	-
ISO 5	100,000	23,700	10,200	3520	832	29
ISO 6	1,000,000	237,000	102,000	35,200	8320	293
ISO 7	-	-	-	352,000	83,200	2930
ISO 8	-	-	-	3,520,000	832,000	29,300
ISO 9	-	-	-	35,200,000	8,320,000	293,000

**Table 3:** Non-viable and viable particle counts. CFU: colony forming units.

Grade	Non-Viable Particle Limits				Viable Particle Limits			
	At Rest		In Operation					
	0.5 µm/m^3^	5 µm/m^3^	0.5 µm/m^3^	5 µm/m^3^	CFU/m^3^	Settling Plates (90 mm) CFU/4 h	Contact Plates (55 mm) CFU/Plate	Footprint (5 Footprint/Glove)
A	3520	20	3520	20	<1	<1	<1	<1
B	3520	29	352,000	2900	10	5	5	5
C	352,000	2900	3,520,000	29,000	100	50	25	-
D	3,520,000	29,000	-	-	200	100	50	-

**Table 4:** Operational qualification (OQ). ISO: International Organization for Standardization.

ISO 4 Classification Assay								
Points	Particle Diameter (µm)							
	0.3 µm		0.5 µm		1 µm		5 µm	
	ft^3^	m^3^	ft^3^	m^3^	ft^3^	m^3^	ft^3^	m^3^
1	1	35	1	35	1	35	0	0
2	11	300	2	71	1	35	0	0
3	0	0	0	0	0	0	0	0
	0.3 µm		0.5 µm		1 µm		5 µm	
**Mean results**	141		35.33		23.33		0	
**FINAL RESULT**	**ISO 4**		**ISO 4**		**ISO 4**		**ISO 4**	
**Positive pressure test**								
**Points**	**Pressure**		**Correction**		**Pressure + Correction**			
1	32.00 Pa		0.00 Pa		32.00 Pa			
2	29.00 Pa		0.00 Pa		29.00 Pa			
3	33.00 Pa		0.00 Pa		33.00 Pa			
4	31.00 Pa		0.00 Pa		31.00 Pa			
5	30.00 Pa		0.00 Pa		30.00 Pa			
**FINAL RESULT: Mean: 31.00 Pa. Positive pressure**								
**Laminar air flow test**								
**Points**	**Laminar flow**		**Correction**		**Laminar flow + Correction**			
1	0.37 m/s		0.01 m/s		0.38 m/s			
2	0.40 m/s		0.02 m/s		0.42 m/s			
3	0.39 m/s		0.01 m/s		0.40 m/s			
4	0.37 m/s		0.01 m/s		0.38 m/s			
5	0.38 m/s		0.02 m/s		0.40 m/s			
6	0.40 m/s		0.02 m/s		0.42 m/s			
7	0.35 m/s		0.01 m/s		0.36 m/s			
8	0.36 m/s		0.02 m/s		0.38 m/s			
**FINAL RESULT: Mean: 0.38 m/s. Laminar flow**								
**Control panel parameters**								
**Week**	**Exhaust filter**		**Downflow filter**		**Inflow**	**Humidity**	**Temperature**	
1	32 Pa		102 Pa		0.46 m/s	32%	20 °C	
2	35 Pa		103 Pa		0.50 m/s	30%	20 °C	
3	32 Pa		103 Pa		0.46 m/s	42%	22 °C	
**FINAL RESULT**	**33 Pa**		**103 Pa**		**0.47 m/s**	**35%**	**21 °C**	
**Week**	**Non-viable particles**		**Viable particles**					
	**0.3 µm**	**0.5 µm**	**Passive air**		**Contact plates**		**Footprint**	
1	0	0	Negative		Negative		Negative	
2	0	0	Negative		Negative		Negative	
3	0	0	Negative		Negative		Negative	
**FINAL RESULT: 0**			**FINAL RESULT: Negative**					

**Table 5:** Performance qualification (PQ).

Control Panel Parameters					
**Month**	**Exhaust Filter**	**Downflow Filter**	**Inflow**	**Humidity**	**Temperature**
1	34 Pa	103 Pa	0.50 m/s	22%	20 °C
2	34 Pa	102 Pa	0.47 m/s	42%	22 °C
3	34 Pa	103 Pa	0.46 m/s	40%	23 °C
4	36 Pa	103 Pa	0.50 m/s	30%	23 °C
**FINAL RESULT**	**35 Pa**	**103 Pa**	**0.48 m/s**	**34%**	**22 °C**
**Month**	**Non-viable particles**		**Viable particles**		
	**0.3 µm**	**0.5 µm**	**Passive air**	**Contact plates**	**Footprint**
1	0	0	Negative	Negative	Negative
2	0	0	Negative	Negative	Negative
3	0	0	Negative	Negative	Negative
4	0	0	Negative	Negative	Negative
**FINAL RESULT: 0**			**FINAL RESULT: Negative**		

**Table 6:** Process quality control.

Process Quality Control						
Batch	Viable Particles			Non-Viable Particles		
	Critical Step 1	Critical Step 2	Critical Step 3	Critical Step 1	Critical Step 2	Critical Step 3
1	Negative	Negative	Negative	0	0	0
2	Negative	Negative	Negative	0	0	0
3	Negative	Negative	Negative	0	0	0
**FINAL RESULT: Negative**				**FINAL RESULT: 0**		

**Table 7:** Product quality control.

Product Quality Control				
Batch	Primary Culture	ATMP		
	p63-Alpha Positive LSC	Number of Cells	p63-Alpha Positive LSC	Microbiological Control
1	72.03%	141,967 cell/cm^2^	82.37%	Negative
2	76.79%	171,504 cell/cm^2^	44.89%	Negative
3	80.31%	104,446 cell/cm^2^	44.55%	Negative

## Data Availability

All the obtained data used to support the findings of this study are available from the corresponding author upon reasonable request.
